# Mitochondrial DNA copy number in Hepatitis C virus-related chronic liver disease: impact of direct-acting antiviral therapy

**DOI:** 10.1038/s41598-023-44665-8

**Published:** 2023-10-26

**Authors:** Amany Elyamany, Rasha Ghazala, Omnia Fayed, Yasmin Hamed, Assem El-Shendidi

**Affiliations:** 1 Department of Internal Medicine (Hepatology Unit), Faculty of Medicine, Alexandria University, Alexandria, Egypt; 2 Department of Medical Biochemistry, Faculty of Medicine, Alexandria University, Alexandria, Egypt; 3 Department of Clinical and Chemical Pathology, Faculty of Medicine, Alexandria University, Alexandria, Egypt

**Keywords:** Microbiology, Molecular biology, Gastroenterology

## Abstract

Hepatitis C virus (HCV) infection can regulate the number and dynamics of mitochondria, and is associated with a prominent hepatic mitochondrial injury. Mitochondrial distress conveys oxidative damage which is implicated in liver disease progression. The present study was conducted to assess the change of mitochondrial DNA (mtDNA) copy number in patients with HCV-related chronic liver disease and the impact of direct-acting antiviral (DAA) therapy. Whole blood mtDNA copy number was measured using real-time quantitative polymerase chain reaction at baseline and 12 weeks after the end of therapy in 50 treatment-naïve HCV-infected patients who achieved sustained viral response (SVR) after DAA therapy and 20 healthy controls. Whole blood mtDNA copy number appeared significantly lower in HCV-infected patients before therapy compared to healthy subjects (*P* < 0.001). Post-treatment, there was significant increase of mtDNA copy number in HCV-infected patients at SVR12 compared to the pre-treatment values (*P* < 0.001), meanwhile it didn’t differ significantly between HCV-infected patients after therapy and healthy subjects (*P* = 0.059). Whole blood mtDNA copy number correlated inversely to the serum bilirubin in HCV-infected patients (*P* = 0.013), however it didn’t correlate significantly to the serum aminotransferases, viral load or fibrosis-4 score (*P* > 0.05). In conclusion, chronic HCV infection has been associated with a prominent mitochondrial injury which could mediate a progressive liver disease. The improved mtDNA content after DAA therapy highlights a possible potential of these drugs to alleviate mitochondrial damage in HCV-related liver disease.

## Introduction

Hepatitis C virus (HCV) is a major cause of liver disease worldwide leading to acute and chronic hepatitis, liver fibrosis, cirrhosis and hepatocellular carcinoma (HCC)^1^. The liver is considered as the metabolic powerhouse of the body, so it is full of mitochondria^2^. Hepatitis viruses can regulate the number, quality and dynamics of the mitochondria, resulting in altered mitochondrial morphology and function^3^. Chronic HCV infection has been associated with a prominent hepatic mitochondrial injury^4^. These effects include mitochondrial swelling and fission, loss of mitochondrial cristae, mitochondrial copy number changes, calcium-mediated mitochondrial depolarization and dysfunction^5^. The severity of HCV infection is represented by the extent of mitochondrial injury^3^.

Mitochondria have the capacity to actively trigger the initiation of a cell intrinsic immune response through induction of interferon (IFN) type I expression via an endogenous ligand, the mitochondrial DNA (mtDNA)^6^. Even though hepatocytes contain hundreds of copies of mtDNA, it is possible that the combination of mtDNA deletions and point mutations could reach a threshold sufficient to induce mitochondrial dysfunction, contributing to the pathogenesis of viral hepatitis^6,7^. Treatment of HCV has evolved with the development of direct-acting antiviral (DAA) drugs which allowed for simplified regimens with increased tolerability and wider therapeutic window, and has radically improved the sustained viral response (SVR) rates even for patients with cirrhosis^8^. Patients who achieved and maintained SVR overtime consequently develop less liver-related complications in comparison with those without SVR^9^. Clinically, the proportions of mtDNA somatic mutations in the liver were linked to the outcomes of IFN-based therapy in chronic viral hepatitis^10^. To date, studies to assess whether SVR after DAA therapy could result in reversal of mitochondrial dysfunction are lacking.

Therefore, the present work was designed to assess the change of mtDNA copy number in patients with HCV-related chronic liver disease and the impact of DAA therapy.

## Materials and methods

### Study population

This prospective study included 50 treatment-naïve patients with chronic HCV infection who were referred to the Hepatology Unit, Department of Internal Medicine at the Main University Hospital in Alexandria, Egypt. Chronic HCV infection was diagnosed on the basis of detectable both serum HCV antibody and viral RNA. The presence of cirrhosis was determined as per the clinical evaluation, laboratory workup, and radiological evidence. Patients with chronic HCV infection were excluded from the study if they had hepatitis B virus (HBV) or human immunodeficiency virus (HIV) co-infection, other causes of chronic liver disease, decompensated chronic liver disease, advanced cardiopulmonary disease, any malignant disease, or pregnancy. Also, 20 age- and sex-matched healthy subjects with no evidence of liver disease were included as a control group.

The study was conducted in accordance with the regulations of the World Medical Association Declaration of Helsinki. The research experiments were approved by the Research Ethics Committee of the Alexandria Faculty of Medicine (Study Approval No.: 0106506-FWA No.: 00018699-IRB No.: 00012098). An informed consent was obtained from all subjects included in the study.

The study participants were evaluated clinically as regards age, sex, and manifestations of chronic liver disease. Routine laboratory investigations included complete blood picture, blood urea and creatinine, liver test profile [serum aspartate aminotransferase (AST), serum alanine aminotransferase (ALT), serum albumin, serum bilirubin, and international normalized ratio (INR)]. In HCV-infected patients, the viral RNA was measured pre-treatment as a baseline, and 12 weeks post-treatment using real-time polymerase chain reaction (RT-PCR) [COBAS Ampliprep/COBAS TaqMan assay, the results were reported in international units per milliliter, and the dynamic range of quantification was 15 to 100,000,000 IU/ml with a claimed lower limit of detection equal to the lower limit of quantification 15 IU/ml]. Virological response was considered when HCV-RNA is below the lower limit of detection at 12 weeks after the end of therapy (SVR12). Fibrosis-4 index (FIB-4) and AST-to-platelet ratio index (APRI) were calculated to assess the stage of hepatic fibrosis non-invasively prior to therapy^11^. Abdominal ultrasonography was used to assess the liver echotexture and size, the presence of cirrhosis, any focal hepatic lesion, and the presence of splenomegaly or ascites. Abdominal ultrasound and serum alpha fetoprotein (AFP) level were checked pre-treatment and monitored biannually through 4 years of follow-up after therapy to detect any HCC that might develop. Eligible HCV-infected patients were offered IFN-free sofosbuvir-based DAA treatment protocol, as per the clinical guidelines for management of HCV infection^12^.

### Measurement of mtDNA copy number

Mitochondrial ND1 gene copy number was measured by real-time quantitative PCR (RT-qPCR) using the nuclear HGB gene as a reference as per the manufacturer’s instructions^13^.

Venous whole blood sample was aseptically obtained from all the study participants and delivered in blood collection tubes [BD Vacutainer] containing K_2_EDTA (di-Potassium ethylene-diamine tetra-acetate) for molecular analysis.

#### Genomic DNA purification

Each blood sample undergone series of reactions to extract cellular DNA [GeneJET Whole Blood Genomic DNA Purification Mini Kit, Catalog Number: K0781, Thermo Fisher Scientific]. Initially, 20 μL of Proteinase K Solution was added to 200 μL of whole blood, mixed by vortexing, then 400 μL of Lysis Solution was added, mixed thoroughly by vortexing or pipetting to obtain a uniform suspension. Then, the sample was incubated at 56 °C for 10 min while vortexing occasionally or by using a shaking water bath, rocking platform or thermomixer until the cells were completely lysed. Then, 200 μL of ethanol (96–100%) was added and mixed by pipetting. Then, the prepared mixture was transferred to the spin column, centrifuged for 1 min at 6000×*g* (~ 8000 rpm). The collection tube containing the flow-through solution was discarded. The column was transferred into a new 2 mL collection tube. Then, 500 μL of Wash Buffer I was added, centrifuged for 1 min at 8000×*g* (~ 10,000 rpm). Then, the flow-through was discarded and the column was placed back into the collection tube. Then, 500 μL of Wash Buffer II was added to the column, centrifuged for 3 min at ≥ 20,000×*g* (≥ 14,000 rpm). Then, the collection tube containing the flow-through solution was discarded and the column was transferred to a sterile 1.5 mL microcentrifuge tube. Then, 200 μL of Elution Buffer was added to the center of the column membrane to elute genomic DNA, incubated for 2 min at room temperature and centrifuged for 1 min at 8000×*g* (~ 10,000 rpm). The purification column was discarded and the purified DNA was stored at − 20 °C.

#### Genomic DNA amplification

Two primer sets were provided to help amplify the mitochondrial ND1 gene, a portion of mtDNA spanning from 3212 to 3300 bp, and the nuclear HGB gene which was used as an internal control [Maxima SYBR Green qPCR Master Mix (2X), Catalog Number: K0251, Thermo Fisher Scientific]. For amplification of the ND1 gene, the pair of primer involved a forward primer (ND1-F): 5′-CCCTAAAACCCGCCACATCT-3′, and a reverse primer (ND1-R): 5′-AGCGATGGTGAGAGCTAAGGT-3′. Meanwhile, for amplification of the HGB gene, the pair of primer involved a forward primer (HGB-F): 5′-GCTTCTGACACAACTGTGTTCACTAGC-3′, and a reverse primer (HGB-R): 5′-CACCAACTTCATCCACGTTCACC-3′. Samples were assayed in duplicates. In each run, DNA sample from a healthy control was included as a calibrator and a negative control. Thermal profile reaction involved an activation step at 95 °C for 10 min, followed by 40-cycles 3-steps cycler conditions of denaturation step at 95 °C for 15 s, an annealing step at 60 °C for 10 min, and an extension step at 72 °C for 30 s. Melting curve was performed to verify specificity and identity of PCR products. Melting curve analysis was built into the software of real-time cyclers. Generally, melting curve data was obtained between 59 and 95 °C. When the temperature was gradually increased, a sharp decrease in SYBR Green fluorescence was observed as the product undergone denaturation. Specific products were distinguished from nonspecific products by the difference in their melting temperatures. To avoid misinterpretition, negative controls were included in all runs. Blank control without DNA was included in each PCR run as a negative control. In order to avoid mispriming or other errors that might happen during PCR, Hot-Start PCR was used. Results were analyzed using the Step One™ Software v2.2. Quantities of mtDNA and nuclear DNA (nDNA) were determined using the relative quantification formula (RQ = 2^–ΔCT^), where ΔCT = CT mtDNA − CT nDNA for every sample is calculated and is used as the exponent of 2 in the equation representing the difference in “corrected” number of cycles to threshold (2^–ΔCT^). Molecular analysis of mtDNA copy number was performed prior to therapy as a baseline and 12 weeks post-treatment, and it was planned to be performed for any patient who might develop HCC during the follow-up period.

### Statistical analysis

Data were analyzed using the Statistical Package for Social Sciences software version 22.0. (SPSS. Armonk, NY: IBM Corporation). Kolmogorov–Smirnov test and Shapiro–Wilk test were used to determine the normality of data distribution. Qualitative variables were mathematically presented as number and percentage. Normally-distributed quantitative variables were mathematically presented as arithmetic mean and standard deviation. Abnormally-distributed quantitative variables were mathematically presented as median and interquartile range. Chi-square (χ^2^) test with Monte Carlo correction or Fisher’s Exact (*FE*) test were used to compare between two groups for qualitative variables as appropriate. Student’s *t*-test was used to compare between two groups for normally-distributed quantitative variables. Mann–Whitney test (*U*) was used to compare between two groups for abnormally-distributed quantitative variables. Pearson’s correlation was used to describe the strength of association between the normally-distributed quantitative variables. Spearman’s correlation was used to describe the strength of association between the abnormally-distributed quantitative variables. Statistical significance was judged at *P* < 0.05 level. All calculated *P* values were two-tailed.

## Results

Baseline clinical and biochemical data of patients included in the study were shown in Table 1. In eligible HCV-infected patients, 46 of the patients (92%) received sofosbuvir + daclatasvir treatment regimen for 12 weeks, while 4 of the patients (8%) received sofosbuvir + daclatasvir + ribavirin treatment regimen for 12 weeks. All HCV-infected patients included in our study achieved SVR at 12 weeks after the end of therapy (SVR12).

**Table 1:** Distribution of baseline clinical and biochemical data in HCV-infected patients and healthy controls.

Parameters	Patients (n = 50)	Healthy controls (n = 20)	Test of significance	*P*
Sex			χ^2^ = 0.333	0.564
Number (male: female)	16:34	5:15		
Percent (male: female)	32%:68%	25%:75%		
Age (years)			*t* = 1.879	0.065
Min.–Max.	21.0 – 70.0	28.0–65.0		
Mean ± SD	50.92 ± 12.48	44.70 ± 12.61		
Hemoglobin concentration (g/dL)	13.45 ± 1.46	13.57 ± 0.86	*t* = 0.417	0.678
Platelets count (× 10^3^/cmm)	222.1 ± 61.36	295.1 ± 43.10	*t* = 4.847*	< 0.001*
Leucocytes count (× 10^3^/cmm)	7.20 ± 1.93	6.45 ± 1.42	*t* = 1.576	0.120
Random blood Glucose (mg/dL)	99.64 ± 35.19	80.50 ± 5.01	*U* = 352.50	0.055
Serum creatinine (mg/dL)	0.75 ± 0.20	0.63 ± 0.18	*U* = 307.50*	0.011*
Alanine aminotransferase (U/L)	58.05 ± 30.71	18.60 ± 5.47	*U* = 30.50*	< 0.001*
Aspartate aminotransferase (U/L)	46.37 ± 20.73	19.50 ± 4.27	*U* = 35.50*	< 0.001*
Serum albumin (g/dL)	3.72 ± 0.32	4.23 ± 0.19	*t* = 6.663*	< 0.001*
Serum bilirubin (mg/dL)	0.53 ± 0.47	0.55 ± 0.19	*U* = 403.0	0.200
International normalized ratio	1.03 ± 0.10	1.0 ± 0.07	*t* = 1.640	0.106
Serum alpha fetoprotein (ng/dL)	6.07 ± 7.03	2.37 ± 0.70	*U* = 372.0	0.096
HCV-RNA (× 10^3^)	969.3 ± 1374.4	NA	–	–
FIB-4	1.55 ± 0.73	NA	–	–
APRI	0.56 ± 0.30	NA	–	–

*HCV* Hepatitis C virus, *RNA* Ribonucleic acid, *FIB-4* Fibrosis-4 index, *APRI* Aspartate aminotransferase-to-platelet ratio index, *NA* Not applicable, *SD* Standard deviation, *t* Student t-test, *U* Mann Whitney test, *χ*^2^ Chi square test, *P* Value of comparison, *Statistical significance at *P* ≤ 0.05.

**Figure 1:**
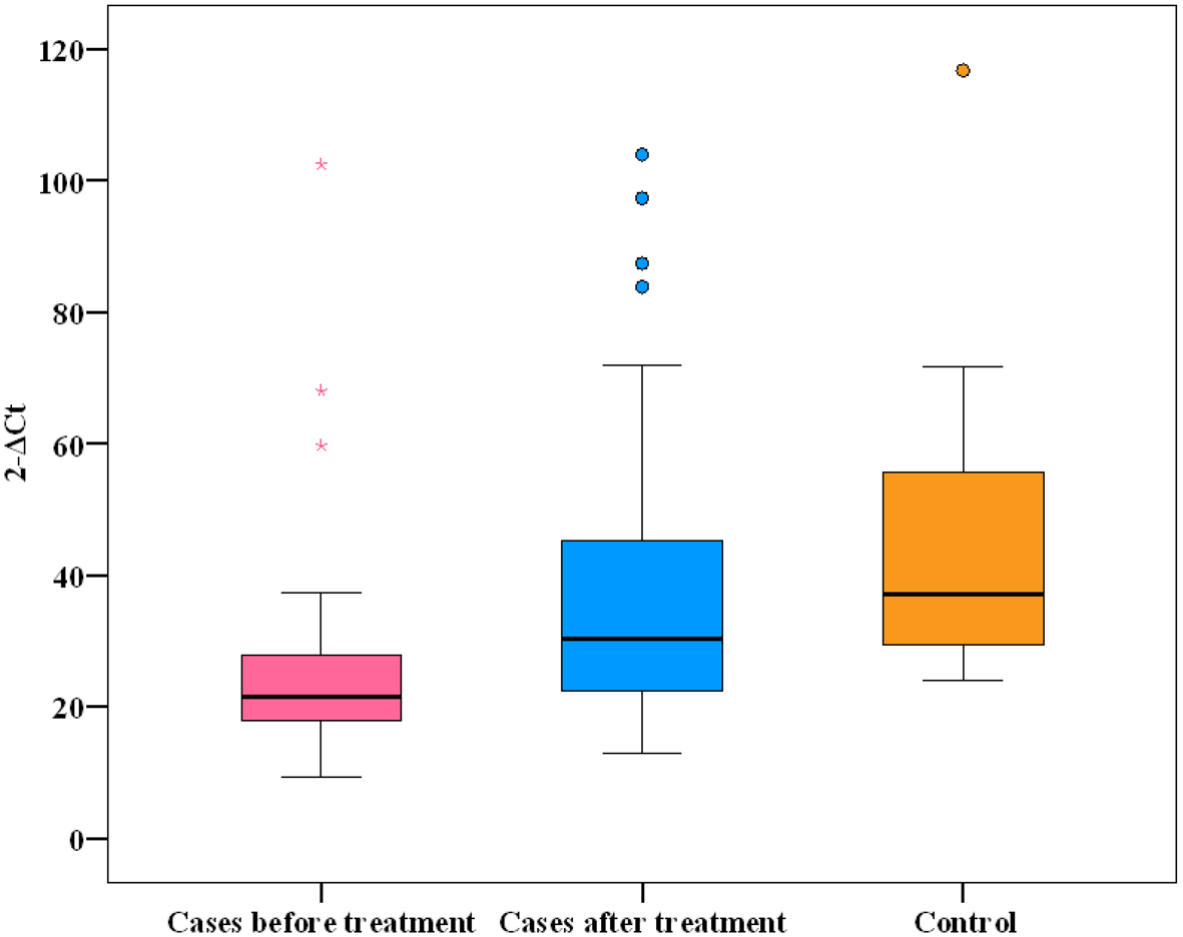
Comparison of whole blood mtDNA copy number (2^−ΔCt^) among HCV-infected patients before therapy, HCV-infected patients after therapy, and healthy controls.

Whole blood mtDNA copy number (2^−ΔCt^) ranged between 9.38 to 102.5 in HCV-infected patients before therapy and ranged between 12.99 to 104.0 at 12 weeks post-treatment, meanwhile it ranged between 24.02 to 116.7 in healthy subjects. Before therapy, the mean mtDNA copy number (2^−ΔCt^) was significantly lower in HCV-infected patients compared to healthy subjects (25.30 ± 15.28 *vs.* 45.05 ± 22.03, *P* < 0.001*). Post-treatment, there was significant increase in the mean mtDNA copy number (2^−ΔCt^) in HCV-infected patients at SVR12 compared to the pre-treatment values (38.07 ± 21.46 *vs.* 25.30 ± 15.28, *P* < 0.001*). Meanwhile, the mean mtDNA copy number (2^−ΔCt^) didn’t differ significantly between HCV-infected patients after therapy and healthy subjects (*P* = 0.059). (Table 2 and Fig. 1).

**Table 2:** Comparison of whole blood mtDNA copy number (2^−ΔCt^) among HCV-infected patients before therapy, HCV-infected patients after therapy, and healthy controls.

Whole blood mtDNA copy number (2^−ΔCt^)	Patients (n = 50)		Healthy controls (n = 20)
	Before therapy	After therapy	
Min.–Max.	9.38–102.5	12.99–104.0	24.02–116.7
Mean ± SD	25.30 ± 15.28	38.07 ± 21.46	45.05 ± 22.03
Median (IQR)	21.55 (17.8–27.9)	30.35 (29.5–55.6)	37.21 (22.5–45.2)
Significance between groups	*P*_1_ < 0.001*, *P*_2_ < 0.001*, *P*_3_ = 0.059		

*mtDNA* Mitochondrial DNA, *SD* Standard deviation, *IQR* Inter quartile range.

*P*_1_: *P* value of Wilcoxon signed ranks test for comparing between HCV-infected patients before and after therapy. *P*_2_: *P* value of Mann Whitney test for comparing between HCV-infected patients before therapy and healthy controls. *P*_3_: *P* value of Mann Whitney test for comparing between HCV-infected patients after therapy and healthy controls.

*Statistical significance at *P* ≤ 0.05.

Whole blood mtDNA copy number (2^−ΔCt^) correlated inversely to serum bilirubin (r = − 0.348, *P* = 0.013*) in HCV-infected patients before therapy, however it didn’t correlate significantly to hemoglobin concentration, platelets count, blood glucose, serum creatinine, serum aminotransferases, serum albumin, INR, serum AFP, HCV viral load, FIB-4 or APRI scores (*P* > 0.05) (Table 3).

**Table 3:** Correlation of whole blood mtDNA copy number (2^−ΔCt^) to some clinical and biochemical variables in HCV-infected patients before therapy.

Parameters	Whole blood mtDNA copy number (2^−ΔCt^) in patients before therapy	
	r_s_	*P*
Age (years)	0.111	0.443
Hemoglobin concentration (g/dL)	− 0.102	0.479
Platelets count (× 10^3^/cmm)	0.151	0.296
Leucocytes count (× 10^3^/cmm)	− 0.168	0.244
Random blood glucose (mg/dL)	0.016	0.912
Serum creatinine (mg/dL)	− 0.071	0.624
Alanine aminotransferase (U/L)	− 0.073	0.612
Aspartate aminotransferase (U/L)	0.017	0.909
Serum albumin (g/dL)	− 0.104	0.471
Serum bilirubin (mg/dL)	− 0.348	0.013*
International normalized ratio	0.178	0.217
Serum alpha fetoprotein (ng/dL)	− 0.166	0.248
HCV-RNA (× 10^3^)	0.063	0.662
FIB-4	0.089	0.540
APRI	− 0.074	0.611

*mtDNA* Mitochondrial DNA, *HCV* Hepatitis C virus, *RNA* Ribonucleic acid, *FIB-4* Fibrosis-4 index, *APRI* Aspartate aminotransferase-to-platelet ratio index, *SD* Standard deviation, *r*_*s*_ Spearman coefficient, *P* Value of correlation, *Statistical significance at *P* ≤ 0.05.

## Discussion

The present study demonstrated a significant decrease in whole blood mtDNA copy number in HCV-infected patients compared to healthy subjects. Similar results were reported by Yen et al.^7^ in a study performed on 132 HCV-infected patients and 47 healthy controls. Also, Zhang and colleagues^14^ conducted a study on 242 patients with chronic HCV infection and 226 control subjects from China and observed a significant decrease in mtDNA copy numbers in CHC patients than in healthy individuals. Inconsistently, Durand et al.^15^ conducted a recent study on 470 HCV-infected patients actively injecting heroin and 260 non-infected heroin users and revealed non-significant difference in mtDNA copy number between drug addicts with CHC and those without HCV infection. This discrepant result may be due to the inclusion of patients with HIV or HBV co-infection or the heroin use which was found to reduce mtDNA copy number independently^16^.

Multiple studies quantified mtDNA using a variety of clinical samples like serum as in Okochi et al.^17^ and Wang et al.^18^, peripheral blood leucocytes (PBL) as in Zhao et al.^19^, whole blood as in Purdue et al.^20^, and sometimes liver tissue as in Yin et al.^21^ and Yamada et al.^22^. In our study, we used whole blood EDTA samples to extract mtDNA, and this can be useful in evaluating large number of cases as it is easily accessible and may be considered a surrogate for the invasive mtDNA quantification in tissues.

It was found that HCV changes the steady-state levels of a mitochondrial protein chaperone, prohibitin, that disturbs the mitochondrial respiratory chain leading to overproduction of reactive oxygen species (ROS)^23^. Induction of oxidative stress in hepatocytes has been assigned to almost all HCV proteins^24^. These viral proteins can induce oxidative stress via induction of nicotinamide adenine dinucleotide phosphate oxidase (NOX)-1/4^25^ and alteration of calcium homeostasis leading to mitochondrial dysfunction^26^. The viral protein NS3/NS4A can cleave the mitochondrial antiviral signaling (MAVS) protein and inhibit mitochondrial antiviral pathway leading to viral persistence^27^. Once ROS give damage to mtDNA beyond a certain threshold, the damaged mtDNA can increase ROS leading to amplification of oxidative stress by encoding deficient subunits for the respiratory chain, which aggravates the oxidative damage to mitochondrial function^28^. Plausibly, the decrease of mtDNA copy numbers in CHC patients represents oxidative damage of mitochondrial nucleic acid.

Several studies confirmed that CHC patients are in a condition of oxidative stress^29–31^. In a study included 75 CHC patients and 22 normal subjects, Barbaro et al.^3^ observed depletion of mtDNA content in the liver tissue of HCV-infected patients and suggested this may represent the expression of a greater impairment of the process of oxidative phosphorylation. They also found a state of oxidative stress, such as depletion of the hepatic and lymphocytic reduced glutathione with increase of the hepatic glutathione disulfide and malonaldehyde, and claimed this may influence the evolution of the liver disease by enhancement of the cytopathic effect of the virus. Also, Cardin et al.^31^ tested the marker of oxidative stress, 8-hydroxydeoxyguanosine, in 110 HCV-infected patients and 20 healthy subjects and found increased its level which was correlated to the serum ALT and the severity of hepatic inflammation.

Viruses other than HCV were found to be associated with increased oxidative stress like HBV, HIV, influenza and other viruses. Chen et al.^32^ conducted a case–control study to test mtDNA content in 76 patients with chronic HBV infection and 96 healthy controls, and found that mtDNA content in the patients was significantly higher than healthy subjects and was negatively correlated to hepatitis B surface antigen. They also showed that the increase of mtDNA content was associated with a decreasing trend of serum ALT and HBV viral load. Regarding HIV infection, Côté et al.^33^ performed a study on 55 HIV-infected patients and 24 healthy subjects and found that mtDNA content was significantly lower in asymptomatic untreated HIV-infected patients than in healthy controls. Also, de Mendoza and colleagues^34^ found that patients with HIV infection had significantly lower mtDNA copy number in PBL sample than HIV-negative controls. Moreover, de Mendoza et al.^35^ demonstrated that there was an inverse relationship between HCV replication and mtDNA content in patients with HIV/HCV co-infection. Additionally, influenza A virus triggered mtDNA release, and dengue virus did that in human lung carcinoma cell line A549 as well^36,37^. Also, herpes simplex virus 1/2 triggered mtDNA stress in mouse embryonic fibroblasts^38^.

The present study demonstrated a significant increase in whole blood mtDNA copy number post-treatment in comparison to pre-treatment values, while it didn’t differ significantly between patients after treatment and healthy subjects. Among our patients, there were only 5 patients (10%) who showed decrease of mtDNA copy number after treatment in comparison to pre-treatment values. All of them had normal radiological image of the liver except for one patient who had coarse hepatic echotexture and high FIB-4 score (2.91), two patients were elderly, one patient had elevated liver enzymes before receiving the treatment (ALT: 115 IU/L and AST: 67 IU/L), and one patient had no obvious issue of interest. All of the 5 patients received the treatment regimen "sofosbuvir + daclatasvir" except for one who received the treatment regimen "sofosbuvir + daclatasvir + ribavirin" due to decreased albumin value (3.3 g/dl)^39^. None of the patients developed HCC after 4 years of post-treatment follow-up as per AFP value and ultrasound surveillance criteria.

To the best of our knowledge, there is only one study that investigated the effect of DAA therapy on mtDNA copy number in human subjects to date. In this study, Durand et al.^15^ found that HCV-infected heroin users who received DAA therapy showed non-significant change of mtDNA copy number post-treatment in comparison to pre-treatment values. This inconsistent result might be due to the presence of HIV and HBV co-infection, co-administration of antiretroviral treatment, and heroin addiction. Meanwhile, Yahya and colleagues^40^ performed a study using the budding yeast exposed to sub-lethal doses of the drugs sofosbuvir and daclatasvir compared with identical cultures grown with chemicals known to induce oxidative stress^41^. The budding yeast was used as a model organism to investigate newly synthesized therapeutics due to simple culture conditions, remarkable genomic homology with humans and easy genetic manipulation^42^. They observed a decrease in mtDNA copy number in yeast cells treated with DAA drugs. This distinct result could be due to temporary compensatory mechanism of the yeast cells aiming to sustain their normal oxidative phosphorylation activity, the absence of HCV infection in the yeast cells, or the different nature of the study subjects.

In the past, Yen et al.^7^ demonstrated increase of mtDNA copy number in PBL sample of HCV-infected patients after IFN-based therapy, and claimed that mtDNA content could be used as a biomarker for treatment effectiveness. Also, Serejo et al.^29^ found that IFN-based therapy in HCV-infected patients promoted inhibition of oxidative stress and was associated with improvement of the hepatic inflammation and fibrosis. Furthermore, de Mendoza et al.^35^ showed that treatment of CHC with pegylated IFN-based therapy reverted the decrease of mtDNA copy number. Also, they found that receiving antiretroviral drugs during the course of anti-HCV therapy in HIV/HCV co-infected patients seemed to increase the mitochondrial damage which was probably because of the harmful synergism between HCV and HIV medications. Interestingly, this deleterious effect was found to be more evident with the drug stavudine as mentioned by de Mendoza and colleagues^34^. They also showed that the treatment of HIV-positive patients with the drug stavudine-containing antiretroviral regimen tended to decrease mtDNA levels even less than drug-naive HIV-positive patients, a finding similar to what was previously reported by Côté and colleagues^33^. Nevertheless, HIV-positive patients on antiretroviral therapy not including the drug stavudine had higher levels of mtDNA in the study of de Mendoza et al.^34^ than what mentioned by Côte and colleagues^33^.

The present study demonstrated that whole blood mtDNA copy number correlated inversely to the serum bilirubin, but not to the serum aminotransferases or the viral load in HCV-infected patients before therapy. Similarly, Zhang et al.^14^ found that mtDNA copy number didn’t correlate to the serum AST level of the total HCV-infected patients, except for male subjects where there was a positive correlation. This result indicated that the relation between AST level and mtDNA content might be affected by gender. Meanwhile, previous studies have linked mtDNA copy number to the inflammatory activity and HCV viremia, as mtDNA copy number was negatively associated with serum AST level, suggesting that it can be used as a biomarker to evaluate the severity of hepatocyte injury^7,43^. This disparity in results may be attributed to the early presentation of cases in our study as all of them were incidentally discovered during a National Health Campaign in Egypt.

Altered mtDNA content was also found to be associated with non-viral causes of liver injury. Sookoian et al.^44^ and Pirola et al*.*^45^ evaluated liver biopsies from patients with non-alcoholic fatty liver disease (NAFLD) and healthy individuals for mtDNA content and observed that mtDNA copy number was significantly lower in the liver of NAFLD patients than the liver of control subjects. In contrast to these results, Kamfar et al*.*^46^ and Chiappini et al.^47^ reported that mtDNA copy number was higher in steatotic liver tissue compared to the liver of normal individuals. One possible explanation for this contradictory result might be the diversity in sample size and ethnic groups. Moreover, several other factors could affect mtDNA copy number like age, sex, comorbidities and drugs, that could be a reason for this discrepancy. Interestingly, Wang and colleagues^18^ conducted a case–control study including 136 cirrhotic HBV cases and 136 non-cirrhotic HBV controls, and found a significantly lower mtDNA content in the serum of cirrhotic HBV patients than that of the non-cirrhotic HBV controls. Moreover, Tang et al.^48^ reported that mtDNA content was remarkably reduced, not only in cirrhotic liver tissues but also in HCC tissues.

Noteworthy, the distressed mitochondria potentially accumulate oxidative damage which is now recognized as a major pro-carcinogenic cofactor in chronic HCV infection^4^. Excitingly, many studies reported that low mtDNA copy number was significantly associated with increased HCC risk, suggesting that mtDNA content might be used as a biomarker to predict HCC occurrence^21,22,49,50^. Zhao and colleagues^49^ conducted a case–control study to assess mtDNA copy number in PBL sample from 274 HBV-related HCC cases, 126 non-cancer patient controls with HBV-related chronic liver disease and 258 healthy controls. They found that there was a significantly lower mtDNA content in HCC cases than in the control subjects. Hashad and colleagues^50^ demonstrated that the lowest mtDNA copy number was noticed among the cases with HCV-related HCC compared to cirrhotic patients and healthy subjects. They suggested a cutoff value of 34 for mtDNA content in order to distinguish between HCV-related HCC and HCV-related cirrhosis. They also claimed this finding might highlight the potential role of mtDNA copy number as a predictor of HCC risk in patients of HCV-related cirrhosis. In our study, 44 patients (88%) showed mtDNA copy number less than the cutoff value of 34 before treatment. While after treatment, there were only 27 patients (54%) with mtDNA copy number below that cutoff value which might be interpreted as a reduction of HCC risk among the treated group. So, we can assume that DAA therapy would reduce the HCC risk possibly due to reversal of HCV-induced oxidative damage, but further studies are needed to support this claim. Various theories were proposed to relate the decrease of mtDNA copy number to HCC development, however the mechanisms for the tumor-specific associations between mtDNA content and the cancer risk remain unclear.

## Conclusion

Based on the results of the present study, the low mtDNA copy number in treatment-naïve HCV-infected patients suggests that chronic HCV infection has been associated with a prominent mitochondrial injury which could be involved in the progression of HCV-related chronic liver disease. The improved mtDNA content after DAA therapy highlights a possible potential of these drugs to alleviate mitochondrial damage in HCV-related liver disease. New strategies to directly manipulate mtDNA and restore the mitochondrial oxidative phosphorylation could be exploited for therapy to reduce the burden of liver disease.

In order to disclose implication of mitochondrial reactivity in the progression of liver disease, it is recommended that the validity of mtDNA copy number as a potential biomarker in relation to cirrhosis and HCC development should be fully explored in prospective clinical trials, with inclusion of patients with other stages of HCV-related liver disease and patients with etiologies of liver disease other than HCV infection would be justified to optimize the present study. Monitoring the evolution of HCV disease after viral cure is extremely important, hence it would be valuable to analyze the effect of treatment-induced viral clearance on mtDNA content in relation to any possible improvement in liver pathology. Moreover, it is imperative to discover the not-yet-known purposeful and off-target effects of DAA therapy as regard the cellular and mitochondrial responses. Indeed the use of paired liver tissues to fully display the mitochondrial content and condition before and after DAA therapy is extremely valuable, but peripheral blood can be easily and repeatedly collected as necessary and certainly exhibit a broader repertoire. Investigation of other ultrastructural and biochemical alterations in mitochondria as well as other markers of oxidative stress would be plausible.

## Data Availability

The datasets presented in the current study are available to editors, reviewers and readers upon reasonable request.
